# Overexpression of the *Prunus sogdiana* NBS-LRR Subgroup Gene *PsoRPM2* Promotes Resistance to the Root-Knot Nematode *Meloidogyne incognita* in Tobacco

**DOI:** 10.3389/fmicb.2017.02113

**Published:** 2017-10-31

**Authors:** Xiang Zhu, Kun Xiao, Haiyang Cui, Jianfang Hu

**Affiliations:** Laboratory of Fruit Physiology and Molecular Biology, China Agricultural University, Beijing, China

**Keywords:** *Prunus sogdiana*, *PsoRPM2* gene, root-knot nematode, HSP90-SGT1-RAR1 co-chaperone, transgenics, *Meloidogyne incognita*

## Abstract

Root-knot nematodes (RKNs), particularly *Meloidogyne incognita*, are the most devastating soil-borne pathogens that significantly affect the production of *Prunus* spp. fruit. RKN infection is difficult to control and consequently causes massive yield losses each year. However, several germplasms of wild *Prunus* spp. have been shown to display resistance to *M. incognita*. Consequently, both the isolation of novel plant resistance (*R*) genes and the characterization of their resistance mechanisms are important strategies for future disease control. R proteins require the co-chaperone protein HSP90-SGT1-RAR1 to achieve correct folding, maturation, and stabilization. Here, we used homologous cloning to isolate the *R* gene *PsoRPM2* from the RKN-resistant species *Prunus sogdiana*. *PsoRPM2* was found to encode a TIR-NB-LRR-type protein and react with significantly elevated *PsoRPM2* expression levels in response to RKN infection. Transient expression assays indicated PsoRPM2 to be located in both the cytoplasm and the nucleus. Four transgenic tobacco lines that heterologously expressed *PsoRPM2* showed enhanced resistance to *M. incognita*. Yeast two-hybrid analysis and bimolecular fluorescence complementation analysis demonstrated that both PsoRAR1 and PsoRPM2 interacted with PsoHSP90-1 and PsoSGT1, but not with one another. These results indicate that the observed PsoRPM2-mediated RKN resistance requires both PsoHSP90-1 and PsoSGT1, further suggesting that PsoRAR1 plays a functionally redundant role in the HSP90-SGT1-RAR1 co-chaperone.

## Introduction

Root-knot nematodes (RKNs) from the genus *Meloidogyne* are a class of sedentary, endozoic plant parasites with a wide host range (Mitkowski and Abawi, 2003). RKNs (particularly *Meloidogyne incognita*) cause significant economic damage in the range of hundreds of billions of dollars annually (Jones et al., 2013). *M. incognita* infected host plants may induce a series of disease resistance reactions that ultimately inhibit both invasion and life cycle completion of nematodes (Abad and Williamson, 2010). The plant immunity response typically consists of two stages: pathogen-associated molecular pattern-triggered immunity (PTI) and effector-triggered immunity (ETI) (Jones and Dangl, 2006). PTI requires pattern recognition receptors (PRRs) located in either the cytomembrane or cytoplasm (Boller and He, 2009; Dodds and Rathjen, 2010). Adaptive pathogens have been reported to interfere with host PTI via small secretory proteins known as effectors (Block et al., 2008; Göhre and Robatzek, 2008; Cunnac et al., 2009). Several plant species have evolved specific resistance genes (*R* genes) that are able to recognize particular effectors, thus facilitating ETI (Zipfel et al., 2006; Tsuda and Katagiri, 2010). Resistance associated with ETI has been shown to be stronger and more enduring than PTI associated resistance (Glazebrook, 2005; Torres et al., 2006; Underwood et al., 2007). *R* gene encoded R proteins play a pivotal role in detecting pathogens and in initiating immune responses (Jacob et al., 2013). Plant R proteins are typically composed of three major domains: a coiled-coil (CC) or a Toll/interleukin-1 receptor (TIR) domain, a nucleotide-binding (NB) domain, and a leucine-rich repeat (LRR) domain (Takken and Goverse, 2012). The *R* gene structure is highly conserved in both plants and mammals (Padmanabhan et al., 2009); however, plants possess considerably more *R* genes, suggesting that plant *R* genes are more pathogen-specific and potentially facilitate more diverse and complex immune responses than those of mammals.

*R* genes for RKNs have been isolated from various plants. The first isolated RKN *R* gene was *Mi-1* (Vos et al., 1998), which originated from tomato (*Solanum lycopersicum*) and conferred race-specific resistance to RKN (*Meloidogyne spp.*) and potato aphids (*Macrosiphum euphorbiae*) (Milligan et al., 1998). Subsequently, RKN *R* genes have either been cloned or mapped from cotton (*Gossypium hirsutum*; *GHNTR1*) (Zhang et al., 2015), potato (*Solanum tuberosum*; *Rmc*) (van der Voort et al., 1999), pepper (*Capsicum annuum*; *Me*) (Celik et al., 2016), and myrobalan (*Prunus cerasifera, Ma*) (Claverie et al., 2011). These RKN *R* genes contain NBS-LRR domains and typically exhibit reduced expression levels in the absence of pathogens. However, RKN *R* gene expression significantly increases in response to RKN infection (Zhang et al., 2015). The plant hypersensitive response (HR) is a disease resistance mechanism that occurs in response to an incompatible plant–pathogen interaction and is typically accompanied by programmed cell death (Karrer et al., 1998). *Mi-1* in tomato induces the HR 12 h after RKN infection, blocking the formation of feeding sites and therefore inhibiting RKN development (Melillo et al., 2006). However, the molecular mechanism underlying *Mi-1*-facilitated RKN resistance remains unclear. Furthermore, due to the species specificity of *R* genes, use of *Mi-1* to engineer RKN resistance in heterologous plants did not result in stable RKN immunity. While transgenic *Mi-1* expressing eggplant (*Solanum melongena*) varieties displayed RKN resistance, no aphid resistance could be found (Goggin et al., 2006). Furthermore, *Mi-1* expressing *Arabidopsis thaliana* and tobacco (*Nicotiana benthamiana*) exhibited no pathogen resistance (Williamson and Kumar, 2006). These results indicate that *R* gene induced resistance signaling pathways vary considerably between different plant species (Martin et al., 2003; Bonardi and Dangl, 2012). Plants with a longer lifespan than herbaceous plants (such as perennial fruit trees) have a higher risk of RKN infection, consequently warranting a more comprehensive, and thus more complex, mechanism of RKN resistance. The identification of novel RKN *R* genes from related plant species may assist in the engineering of stable and enduring RKN resistance.

Studies using model plant species revealed that R proteins require the co-chaperone protein complex HSP90-SGT1-RAR1 to achieve correct folding, maturation, and stabilization (Huang et al., 2014). The heat shock protein 90 (HSP90) is a highly conserved protein in higher plants, is a suppressor of the G2 allele of *skp1* (SGT1), and is required for *mla12* resistance (RAR1) (Kadota and Shirasu, 2012). HSP90 is involved in the assembly, maturity, and stabilization of numerous critical signaling proteins in eukaryotic cells, and typically exists as a homodimer (Csermely et al., 1998; Hubert et al., 2003). SGT1 regulates important biochemical processes, such as ubiquitination and kinetochore assembly, and maintains the stability of the R protein structure in the absence of pathogens (Austin et al., 2002). RAR1 was first characterized in barley (*Hordeum vulgare*), and while *rar1* mutant plants appear morphologically normal, they are less resistant to fungal pathogens, despite the presence of the *R* gene *MLA12* (Bieri et al., 2004). Both HSP90 homodimer and SGT1 interact with a number of R proteins, and consequently, loss of function of HSP90, SGT1, or RAR1 will reduce the functionality of R proteins such as MLA1, MAL6, Rx, RPM1, RPS5, Mi-1, and I-2, thus compromising plant immunity (Lu et al., 2003; Bieri et al., 2004; Holt et al., 2005; Bhattarai et al., 2007; Botër et al., 2007; Van Ooijen et al., 2010). Recently, the relationship between the HSP90-SGT1-RAR1 co-chaperone protein complex and RKN resistance has been further characterized. While RKN resistance is not affected in *Mi-1*-engineered tomato in response to RNA interference induced reduction of *SlRAR1* gene expression, reduced expression of *SlHSP90* and *SlSGT1* results in increased numbers of galls and egg mass in plants exposed to *M. javanica* RKNs (Bhattarai et al., 2007). This suggests that HSP90 and SGT1 also have a function in RKN resistance. Most studies that examine the relationship between *R* genes and the HSP90-SGT1-RAR1 co-chaperone protein have been based on herbaceous model plant species, and comparable research for woody plants is still missing.

The Xinjiang wild myrobalan (*Prunus sogdiana*) is distributed throughout native forests in the eastern region of the Tianshan Mountains (Esmenjaud et al., 1996; Wang et al., 2006; Kang et al., 2008). We collected *P. sogdiana* seeds from the Xinjiang Yili native forest in 2005 and used these seeds from 2007 onward to cultivate more than 150 individual *P. sogdiana* plants at the Shangzhuang experimental station of the China Agricultural University (CAU). Among the resulting plants, we found several individuals that displayed complete resistance to *M. incognita* and repeated this experiment six times, which lasted 6 years (Xiao et al., 2010; Li et al., 2011; Liu et al., 2013; Chen et al., 2015; Qiu et al., 2016). Via homologous cloning of genes from highly resistant *P. sogdiana* individuals based on reported RKN *R* gene sequences, we identified a series of NB domain fragments that respond to RKN invasion. Full-length *R* gene cDNA sequences were obtained via 3′- and 5′- RACE, revealing *PsoRPM2* as the underlying *R* gene. To characterize the observed *M. incognita* resistance conferred by this gene, we created transgenic heterologously expressing *PsoRPM2* lines of the RKN-susceptible tobacco variety W38 (*Nicotiana tabacum* cv. W38). Analyses of the interactions between PsoRPM2, PsoHSP90-1, and PsoSGT1 provided insight into the underlying mechanism of *P. sogdiana* RKN immunity.

## Materials and Methods

### Plant and Nematode Materials

*Prunus sogdiana* seedlings were planted at the Shangzhuang test station at the CAU. Following 5 years of evaluation, individual *P. sogdiana* plants were determined to be either highly resistant or susceptible to *M. incognita* and were used to source root tissue for hardwood cutting and RNA extraction.

The hardwood cutting experiment was performed from 2014 to 2015 using both resistant and susceptible plant individuals. Fifteen resistant and fifteen susceptible hardwood cuttings with heights between 20 and 30 cm and that displayed uniform growth were inoculated with 2000 *M. incognita* J2 nematodes per seedling. Five resistant and five susceptible seedlings were irrigated with identical amounts of water to serve as experimental controls. All of the root tips were collected at 0, 1, 3, and 5 days post-infection (dpi) for gene cloning and expression assays.

Seedlings of tobacco W38 (*Nicotiana tabacum* cv. W38) and *Nicotiana benthamiana* were cultured in a greenhouse. *M. incognita* nematodes were sourced from the laboratory of Jian Heng from the Institute of Plant Protection of the CAU. Nematode cultures were maintained according to published method (Priya et al., 2011) with slight modifications: eggs were collected from susceptible tobacco W38 roots, placed on nylon netting floating in water, and maintained in darkness at 30°C for 5 days, at which point juvenile nematodes (J2) were collected for analysis.

### Gene Cloning and Expression Assays

#### RNA Extraction and cDNA Synthesis

Total RNA was extracted via the cetyltrimethylammonium bromide (CTAB) method from both resistant and susceptible myrobalans root tips at 0, 1, 3, and 5 dpi. cDNA was synthesized using a reverse transcription system (Promega, United States) and was used to either clone typical resistance gene analogs (RGAs) or for quantitative reverse transcription PCR (qRT-PCR) analysis (using the machine model ABIPRISM 7500).

#### PCR Amplification from cDNA to Clone RGAs

To obtain the typical RGAs for RKN-resistant *P. sogdiana*, cDNA was synthesized from the RNA of roots that have been collected at 1 dpi and from a single *P. sogdiana-*resistant plant. The RGA primer pair MP-F/MP-R (see Supplementary Table S1) was designed based on the consistently conserved P-loop sequence of NBS domains among known resistance R proteins; particularly Mi-1, Ma (resistant alleles), GHNTR1, and PkMi proteins, as previously described (Li et al., 2011). The utilized PCR program has previously been described (Bouktila et al., 2014). RGA fragments were connected with a TA-cloning Kit (Tiangen) and then sequenced. The amino acid sequences of all RGA fragments were predicted using BioXM 2.6 and sequences with continuous open reading frame (ORF) were chosen for querying databases via NCBI-BLAST^1^. Sequences with a predicted P-loop motif were selected as candidate *R* genes for further study. All primers were designed using the software package Primer 5.0.

#### Determining the Full-Length Sequence of *PsoRPM2* via RACE and Expression Profiling

To obtain full-length sequences for candidate *R* genes from amplified RGA fragments, primers were designed for 5′- and 3′-rapid amplification of cDNA ends (RACE) PCR (see Supplementary Table S1). RACE was performed following previously described methods (Yeku and Frohman, 2011). The DNAMAN5.0 software was used to analyze the full-length cDNA sequence of the novel *R* gene and the predicted protein domains of this novel sequence were analyzed via NCBI-BLAST. The NB-ARC domain of the novel R protein was identified via the Phyre2 program^2^ (Kelley et al., 2015). The phylogenetic tree of the R protein was created in MEGA 5.1. All primers used for expression analysis are listed in Supplementary Table S1. The primer pair to target the coding sequence (CDS) of *PsoRPM2* was designed to clone and compare alleles in both resistant and susceptible *P. sogdiana* plants (see Supplementary Table S1). qRT-PCR primers were designed to analyze the *PsoRPM2* expression in resistant and susceptible plants following RKN infection (see Supplementary Table S1). qRT-PCR was performed via the SuperReal PreMix Plus (Tiangen Biotech Co., Ltd., China) and an Applied Biosystems 7500 instrument (Thermo Fisher Scientific, United States), with 40 cycles of 95°C for 10 s followed by 60°C for 30 s. Relative expression levels were calculated using the 2^-ΔΔC_t_^ method, the *RPII* gene was used as reference gene, and the primers referred to Tong et al. (2009).

#### Cloning of Co-chaperonin Genes

Homology-based cloning was used to clone genes that encoded members of the chaperonin complex (*HSP90, SGT1*, and *RAR1*) from a single resistant *P. sogdiana* plant. *HSP90* typically has several homologues in plant genomes. Based on the genomic data of the related species *Prunus mume*^3^, three *HSP90* candidate genes were identified after NCBI-BLAST database searches using the CDS of *SlHSP90-1* (GeneID: 543902), *HSP90-1* (GeneID: 103342005), *HSP90-2* (Gene ID: 103319045), and *HSP83* (GeneID: 103333719) as queries. In the genome of *P*. *mume, PmSGT1* and *PmRAR1* were selected as suitable candidates. This was done because they were both present in a single copy and displayed high-level conservation among higher plant genomes. The CDSs of *PmSGT1* (GeneID: 103333704) and *PmRAR1* (GeneID: 103333306) were identified after NCBI-BLAST database searches using *SlSGT1* (GeneID: 101247681) and *AtRAR1* (GenBank: AF192262.1) as queries, respectively. The primers that were designed for the cloning of these five genes are listed in Supplementary Table S1. All sequencing was performed and all primers were produced by the Taihe Biotechnology Co., Ltd. (Beijing).

### Plasmid Construction

To isolate *PsoRPM2*, the BioXM 2.6 software package was used to analyze the restriction enzyme cutting sites within the *PsoRPM2* CDS. Subsequently, the *PsoRPM2* CDS was PCR amplified using primers designed via Primer 5.0 (see Supplementary Table S1). *PsoRPM2* was then inserted between the NcoI and SpeI sites within the pCAMBIA 1305.1 plant expression vector, creating pCAMBIA 1305.1-35S-*PsoRPM2*-GFP. The T4 DNA ligase (TAKARA) was used to combine DNA fragments and sequencing was performed to verify vectors. pCAMBIA 1305.1-35S-GFP was selected as the negative control. All bacterial transformations during the cloning process were performed using the freeze-thaw method (Weigel and Glazebrook, 2006).

To create vectors for the protein interaction analysis, the internal restriction sites within *PsoRPM2, PsoSGT1* (GeneBank: KY225327), *PsoRAR1* (GeneBank: KY225328), *PsoHSP90-1* (GeneBank: KY225329), *PsoHSP90-2* (GeneBank: KY225330), and *PsoHSP90-83* (GeneBank: KY225331) CDSs were determined via BioXM 2.6. Gene-specific primers that contained appropriate restriction sites (see Supplementary Table S1) were designed to both amplify and clone genes into expression vectors. The pGADT7 and pGBKT7 vectors described in the Yeastmaker^TM^ Yeast Transformation User Manual (Clontech) were used for a preliminary screen for candidate proteins that interacted with PsoHSP90 and were also used for *in vitro* protein interaction experiments. The vectors pCAMBIA1300-YFPn and pCAMBIA1300-YFPc were used to construct BiFC vectors that were used to confirm potential protein interactions *in vivo*. The primers and restriction enzymes used for this purpose are listed in Supplementary Table S1.

### Protein Location Analysis of PsoRPM2

To further demonstrate the biological characteristics of the PsoRPM2 protein, the subcellular localization was analyzed. The transient transformation was performed following the procedure published by Sparkes et al. (2006). *Agrobacterium tumefaciens* liquid with reconstruction vectors was injected into 4-week-old *N. benthamiana* plants using a needleless syringe (1 ml). Three days after the injection, the infected leaf epidermis was analyzed using an Olympus BX61 fluorescence microscope (Olympus Fluo View FV1000).

### Generation of Transgenic Tobacco Lines

The generated pCAMBIA 1305.1-35S-PsoRPM2-GFP vector was transformed into the *A. tumefaciens* strain EHA105. Cultures of the resulting transformed *A. tumefaciens* strain were infiltrated into leaves of *N. benthamiana*. Agroinfiltrated tobacco W38 leaves were placed on solid MS medium (1× MS, 2.0 μg/ml 6-BA, 0.5 μg/ml IAA, and 50 mg/ml Cef) and maintained in darkness. After 3 days, transformed leaves were subcultured in fresh antibiotic-containing MS (1× MS, 2.0 μg/ml 6-BA, 0.5 μg/ml IAA, and 50 mg/ml Cef) and maintained under a 16 h/8 h light/dark regime at 26°C. The *PsoRPM2* tobacco transformants were selected via supplementing the antibiotic-containing MS with rifampicin (50 μg/ml). To detect the transgenic gene expression, gene-specific primers were designed for *HYGROMYCIN PHOSPHOTRANSFERASE* (*HPT*) (Suzuki et al., 2001) and *PsoRPM2* (see Supplementary Table S1). RT-PCR amplicons corresponding to *PsoRPM2* and *HPT* expression were observed in agarose gels and then purified and confirmed via sequencing. Several confirmed *PsoRPM2* and *GFP* (negative control) transformants were then transplanted into culture bottles to create biological replicates.

### RKN Inoculation and Resistance Assay

RKN infection assays were performed as previously described (Niu et al., 2015), with slight variation: To evaluate resistance conferred by *M. incognita PsoRPM2* in tobacco, 2,000 nematode J2s (suspended in liquid) were infiltrated into the soil surrounding the roots of each tobacco plant. Thus, infected plants were cultivated in a greenhouse at 18–25°C. At 45 dpi, the roots were removed from the soil and cleaned; then, previously published methods (Vos et al., 1998) were utilized to quantify root weight, total number of roots, number of roots with galls, number of galls, and egg mass. To assay the expression level of PsoRPM2 in root of T1 tobaccos, qRT-PCR were used to test the root tips of T1 tobaccos 0, 1, 3, and 5 dpi with 2000 nematode J2s. *Ntactin* were used as reference gene and the qRT-PCR primers were consulted Duan et al. (2016). All statistical analyses and data summaries were obtained with the PASW Statistics 18 software.

### Yeast Two-Hybrid Analysis

Yeast two-hybrid analysis was conducted as previously described (Gu et al., 2015) using the yeast strain AH109, synthetic dropout minimal medium, and β-galactosidase activity assay reagents according to the Yeastmaker^TM^ Yeast Transformation User Manual (Clontech). All restriction enzyme sites used to create the pGBKT7 or pGADT7 vectors are listed in Supplementary Table S1. Competent yeast cells were transformed with specific vector combinations using the freeze-thaw method. The AH109 yeast strain was grown on SD/-Leu-Trp medium for 4–5 days at 30°C, and 10 independent clones were picked and cultured on SD/-Ade-His-Leu-Trp medium at 30°C for 3–4 days. Then, they were analyzed for β-galactosidase activity via color development using α-X-Gal (4 mg/ml, 1–2 μl per colony). Primer used to construct victors were listed in Supplementary Table S1.

### BiFC Analysis

Cultures of *Agrobacterium* strains that carried individual vectors (pCAMBIA 1300-35S-PsoHSP90-1-nYFP, pCAMBIA 1300-35S-PsoHSP90-1-cYFP, pCAMBIA 1300-35S-SGT1-cYFP, pCAMBIA 1300-35S-PsoRPM2-nYFP, pCAMBIA 1300-35S-PsoRAR1-nYFP and pCAMBIA 1300-35S-PsoRAR1-cYFP) were paired and co-infiltrated into healthy leaves of greenhouse-cultured *N. benthamiana* plants (Sparkes et al., 2006). The combination pCAMBIA 1300-35S-PsoRPM2-nYFP and pCAMBIA 1300-35S-PsoRAR1-cYFP was used as a negative control. Related primers see Supplementary Table S1. Five days after infiltration, the transformed leaf epidermis was analyzed via an Olympus BX61 fluorescence microscope (Olympus Fluo View FV1000).

## Results

### *PsoRPM2* Gene Cloning and Protein Analysis

**FIGURE 1:**
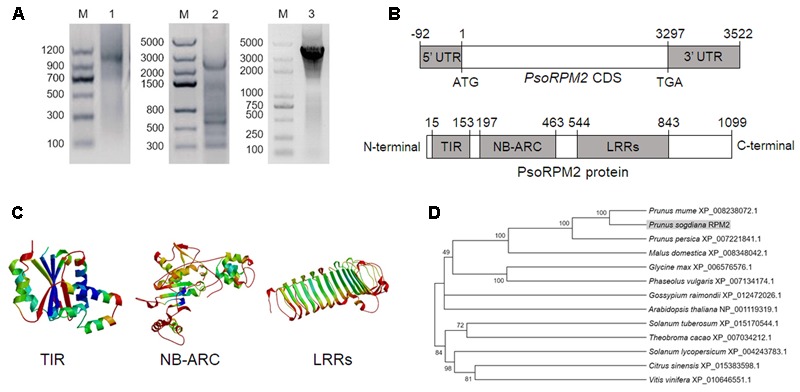
*PsoRPM2* cloning and protein analysis. **(A)** The mRNA clone of *PsoRPM2* generated by 3′-RACE (lane 1), 5′-RACE (lane 2), and full-length amplification (lane 3). M: DNA marker. **(B)** Schematic representations of *PsoRPM2*. **(C)** The predicted 3-dimensional structures of the TIR, NB-ARC, and LRR domains within PsoRPM2. **(D)** Phylogenetic tree containing PsoRPM2 (highlighted) and homologous proteins from other related plant species.

PCR amplification with primers that were designed to identify RGAs and root tissue from RKN-resistant *P. sogdiana* plants detected a single major amplicon with a size of approximately 520 bp. This fragment was cloned via TA-cloning and then sequenced. Protein BLAST analysis with the resulting sequence indicated it to be a partial NBS-ARC domain. To obtain the full-length nucleotide sequence of the amplified fragment, 3′- and 5′-RACE were utilized to amplify a 1000-bp sequence and a 2500-bp sequence, respectively. Following sequencing and splicing, the 3614-bp full-length target gene mRNA sequence was named *P. sogdiana RESIST PATHOGEN M. incognita (PsoRPM2*; GenBank: KU198632.1; **Figure 1A**). The CDS of *PsoRPM2* had a length of 3297 bp and was flanked by a 92-bp 3′-UTR and a 225-bp 5′-UTR. Genomic DNA sequencing revealed no introns within *PsoRPM2*, and PsoRPM2 was found to contain a TIR domain (139 aa), a NB-ARC domain (267 aa), and a LRR domain (300 aa) (**Figure 1B**). The TIR domain contained three conserved motifs, the NBS-ARC domain contained a P-loop and kinases 1, 2, and 3 motifs, and the LRR domain contained 13 leucine-rich repeats (**Figure 1C**). The CDS regions of *PsoRPM2* genes in resistance and susceptible *P. sogdiana* plants were identical. Phylogenetic analysis suggests that PsoRPM2 was closely related to homologous proteins of *Prunus mume, Prunus persica*, and *Malus domestica* (**Figure 1D**).

### *PsoRPM2* Expression and PsoRPM2 Subcellular Localization

**FIGURE 2:**
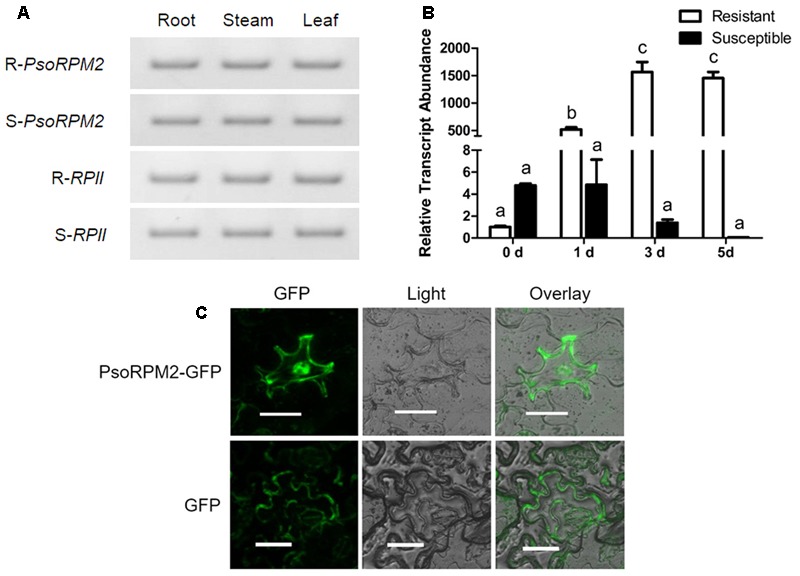
*PsoRPM2* expression profile and subcellular localization of PsoRPM2 protein. **(A)** *PsoRPM2* expression in various tissues of resistant (upper two panels) and susceptible (under two panels) *P. sogdiana* plants. *RPII* was used as a reference gene. **(B)** *PsoRPM2* expression in root tips following *M. incognita* infection measured using qRT-PCR. The results show the means ± SD based on three biologic repetitions, each with three technical repetitions. Different letters denote a significant difference at *p* < 0.05 (Tukey’s test). **(C)** Protein localization analysis showing PsoRPM2-GFP in the cytoplasm and nucleus of *N. benthamiana* epidermal cells. Bar = 40 μm.

Analysis of the tissue expression profile demonstrated that *PsoRPM2* was expressed at comparable levels in root, stem, and leaf tissues both in resistant and susceptible plants of *P. sogdiana* (**Figure 2A**). However, the *PsoRPM2* expression levels observed during *M. incognita* infection differed significantly between resistant and susceptible plants (**Figure 2B**). Prior to RKN inoculation (0 dpi), *PsoRPM2* was expressed at a significantly higher level in susceptible plants than in resistant plants in comparison to the reference gene *RPII* (*p* < 0.05, Tukey’s test). At 1 dpi, *PsoRPM2* expression in resistant plants rapidly increased to an approximately 500-fold higher level than that observed at 0 dpi, while the level of *PsoRPM2* expression in susceptible plants remained unchanged. At 3 dpi, the *PsoRPM2* expression level in resistant plants had still increased and the elevated expression persisted until 5 dpi, while the *PsoRPM2* expression in susceptible plants decreased after 1 dpi reaching a level that was difficult to detect at 5 dpi (**Figure 2B**). An analysis of transiently expressing *PsoRPM2-GFP N. benthamiana* leaf epidermal cells indicated that PsoRPM2 was localized in both the cytoplasm and the nucleus (**Figure 2C**).

### Overexpression of *PsoRPM2* in Tobacco Enhances RKN Resistance

**FIGURE 3:**
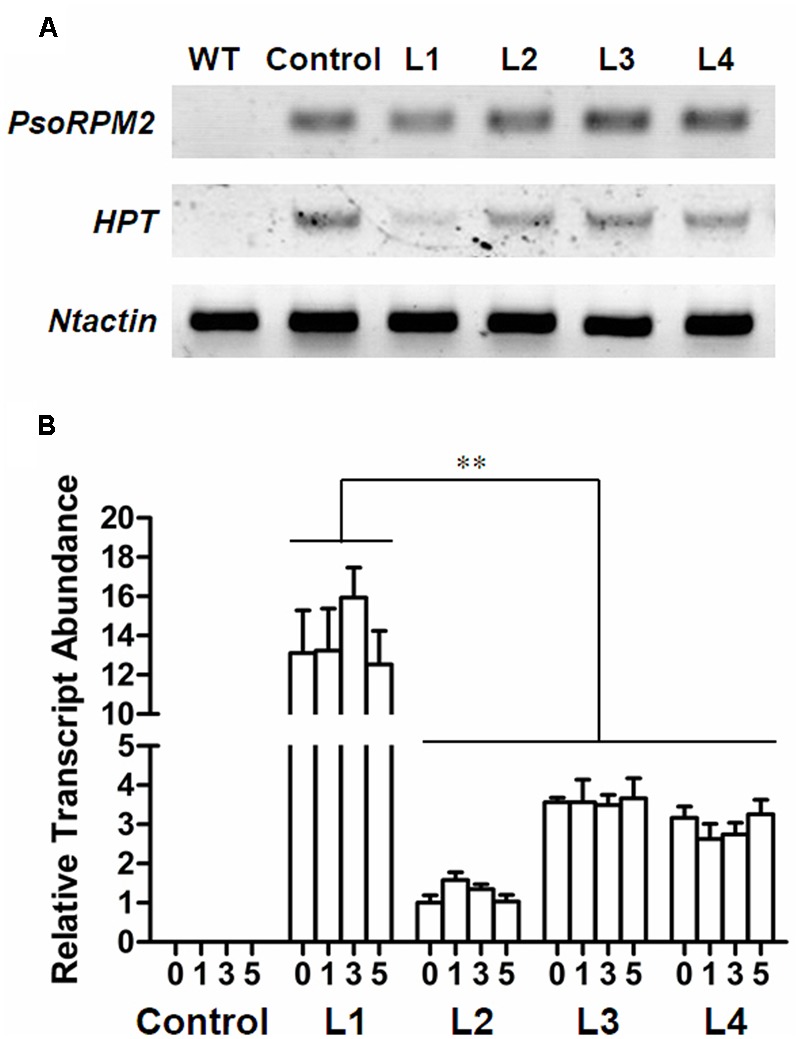
Expression of exogenous genes in transgenic tobacco lines. **(A)** Detection of *PsoRPM2, HPT* and *Ntactin* gene in transgenic W38 tobacco lines, the *PsoRPM2* gene were positive in L1-4, *HPT* gene were positive in L1-4 and negative control, *Ntactin* was used as reference gene. To analyze each sample, 28 cycles were proceeded in RT-PCR. **(B)** Expression of *PsoRPM2* gene in T1 plants inoculated. The abscissa 0, 1, 3, and 5 indicates the dpi with 2000 *M. incognita* J2 nematodes per seedling. The results represent the means ± SD based on three biologic repetitions, each with three technical repetitions. ^∗∗^Means extremely significantly different (each line, *n* = 3, *p* < 0.01, Tukey’s test).

We transferred *PsoRPM2* into the RKN susceptible tobacco variety W38. Four resulting transgenic lines (L1, L2, L3, and L4) and control line (transmitted empty-vector) were selected by testing the *HPT, PsoRPM2* and *Ntactin* in **Figure 3A**. The *PsoRPM2* expression in L1-4 and control after RKN inoculation in 0, 1, 3, and 5 days were also examined by qRT-PCR. The expression profile of *PsoRPM2* in transgenic L1-4 has no obvious difference between 0, 1, 3, and 5 dpi, L1 has the highest level compared with other three lines (*p* < 0.01) (**Figure 3B**).

**FIGURE 4:**
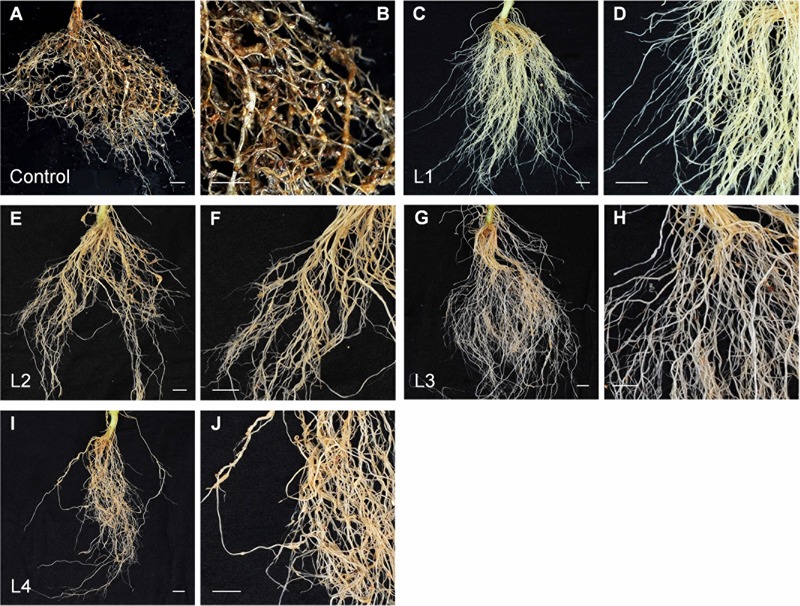
Total mass and magnified images of RKN-infected roots from wild-type and *PsoRPM2*-expressing transgenic tobacco lines. **(A,B)** Total mass **(A)** and magnified detail **(B)** of RKN-infected roots from transgenic W38 tobacco expressing GFP, included as a negative control. **(C–J)** Total mass **(C,E,G,I)** and magnified detail **(D,F,H,J)** of RKN-infected roots from PsoRPM2-expressing transgenic tobacco lines 1 **(C,D)**, 2 **(E,F)**, 3 **(G,H)**, and 4 **(I,J)**. Photographs were taken at 45 dpi.

A RKN resistance assay was performed using 2,000 *M. incognita* J2s per plant and the resulting tobacco roots were photographed at 45 dpi (**Figures 4A–J**). Control roots exhibited a dense distributed of many galls throughout the root mass, and multiple galls could be found on each root (**Figure 4A**). Most galls observed on control roots formed an egg mass, consisting of many RKN eggs (**Figure 4B**). The roots of the first transgenic *PsoRPM2*-expressing tobacco line (L1) displayed robust growth with a minimal number of galls and a complete lack of egg mass formation (**Figures 4C,D**). Roots of the second transgenic line (L2) displayed some galls, but fewer than in control roots (**Figures 4E,F**). Roots of the third (L3; **Figures 4G,H**) and fourth (L4; **Figures 4I,J**) transgenic lines displayed a higher gall density and egg mass production than L1, but a lower gall density and egg mass production than L2.

**FIGURE 5:**
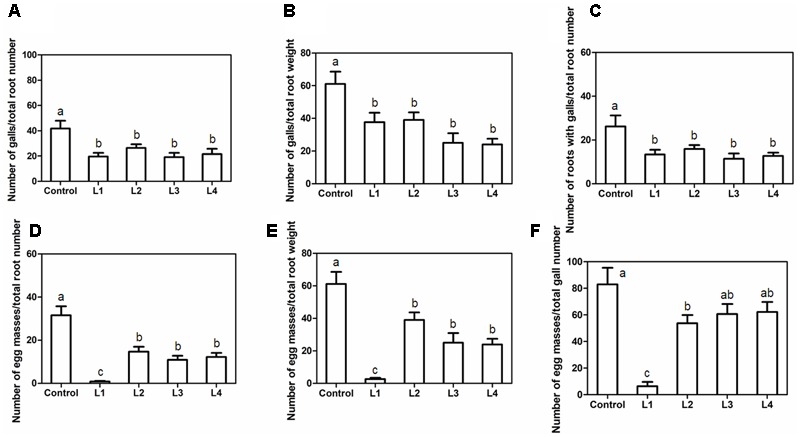
RKN resistance conferred by *PsoRPM2* expression in T1 plants. **(A)** Number of galls/total root number. **(B)** Number of galls/total root weight. **(C)** Number of roots with galls/total root number. **(D)** Number of egg masses/total root number. **(E)** Number of egg masses/total root weight. **(F)** Number of egg masses/total gall number. The results show the means ± SD. (Control, *n* = 9; line 1, *n* = 11; line 2, *n* = 8; line 3, *n* = 9; line 4, *n* = 6). Different letters denote a significant difference at *p* < 0.05 (Tukey’s test).

To assess whether in planta *PsoRPM2* expression conferred resistance to *M. incognita*, we inoculated L1, L2, L3, and L4 with 2,000 *M. incognita* J2s per plant. Compared to control plants, no apparent morphological differences were observed in the resulting transgenic plants. At 45 dpi, the average gall number was reduced both per total root number and per total root weight (**Figures 5A,B**), and the proportion of root mass that exhibited galls was significantly lower in transgenic lines than in the control (**Figure 5C**). In agreement with these findings, the average egg mass number was decreased both per total root number and per total root weight (**Figures 5D,E**), and the proportion of galls with egg mass was significantly lower in all transgenic lines than in the control (**Figure 5F**).

### PsoRPM2 Interacts with PsoHSP90-1 and PsoSGT1 and Is Essential for RKN Resistance

**FIGURE 6:**
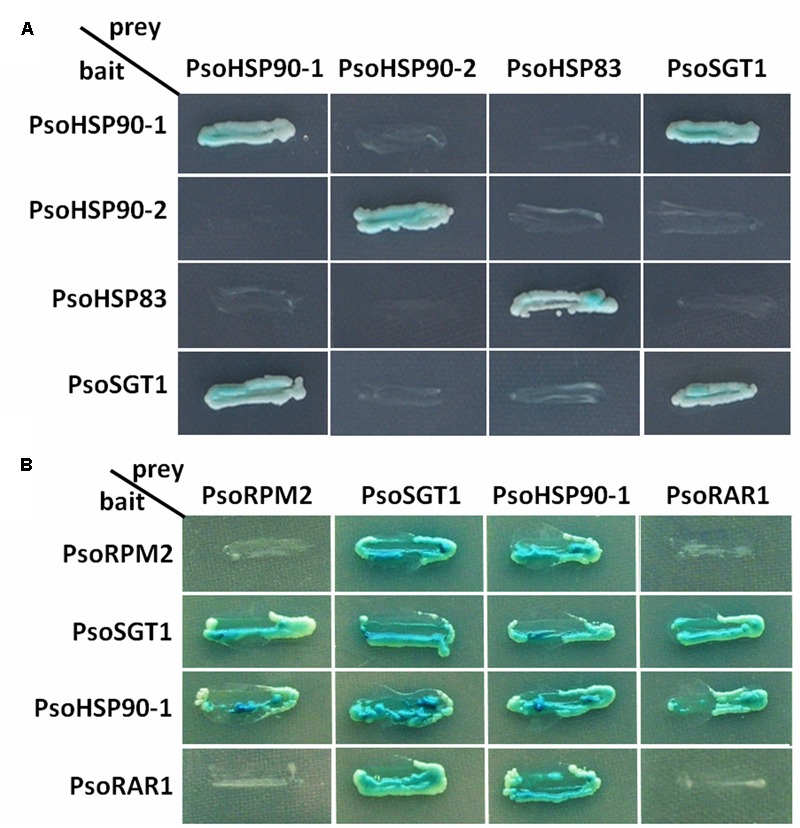
Analysis of interactions between PsoRPM2 and PsoHSP90-1, and PsoSGT1 and PsoRAR1 using the yeast two-hybrid system. β-Galactosidase activity was detected using X-α-Gal as a substrate. **(A)** Selection of candidate PsoSGT1-interacting PsoHSP90 proteins. **(B)** Interactions between PsoRPM2 and its chaperonin proteins.

Since the RKN-resistance of *PsoRPM2* transgenic tobacco lines was enhanced and since the PsoRPM2 protein could be located in the cytoplasm, this suggests that PsoRPM2 might be synergistic with the HSP90-SGT1-RAR1 co-chaperone complex in *P. sogdiana* similar to herbaceous model plants. Thus, we designed a yeast two-hybrid test to analyze the interaction between the homologous genes *PsoHSP90, PsoSGT1*, and *PsoRAR1*, which are three members of the co-chaperone protein complex from *P. sogdiana*. Three *HSP90* homologous genes were cloned from Xinjiang wild *P. sogdiana* via considering the genomic data of *P. mume* genome: *PsoHSP90-1* (GeneBank: KY225329), *PsoHSP90-2* (GeneBank: KY225330), and *PsoHSP83* (GeneBank: KY225331); one *SGT1* homologous gene, *PsoSGT1* (GeneBank: KY225327); and one *RAR1* homologous gene, *PsoRAR1* (GeneBank: KY225328). The Yeast two-hybrid experiments showed that PsoHSP90-1, PsoHSP90-2, PsoHSP83, and PsoSGT1 proteins were able to grow on the 4-defect medium and could be blue-tinted via X-gal, indicating that they could from homodimers. PsoHSP90-1 did not interact with PsoHSP90-2 or PsoHSP83, and only PsoHSP90-1 interacted with PsoSGT1. No interaction was found between PsoHSP90-2 and PsoHSP83 with PsoSGT1 (**Figure 6A**). Considering that the same phenomenon had occurred in *Arabidopsis* (Takahashi et al., 2003), we suggest that PsoHSP90-1 may be involved in the formation of an immune-related co-chaperone protein complex in *P. sogdiana*. Thus, PsoHSP90-1 was selected for further analysis.

In the subsequent interaction tests, yeast two-hybrid assays of PsoRPM2, PsoHSP90-1, PsoSGT1, and PsoRAR1 were used to test their relationship. In **Figure 6B**, the protein PsoRPM2 is shown to interact with PsoHSP90-1 and PsoSGT1, both as bait and target protein; however, no interactions of the PsoRPM2 protein itself or with the PsoRAR1 protein were found. The PsoSGT1 protein could interact with PsoRPM2, PsoHSP90-1, and PsoRAR1. The PsoHSP90-1 protein also interacted with all four proteins. However, the PsoRAR1 protein only interacted with PsoSGT1 and PsoHSP90-1, while PsoRAR1 itself or with the PsoRPM2 protein did not interact (**Figure 6B**).

**FIGURE 7:**
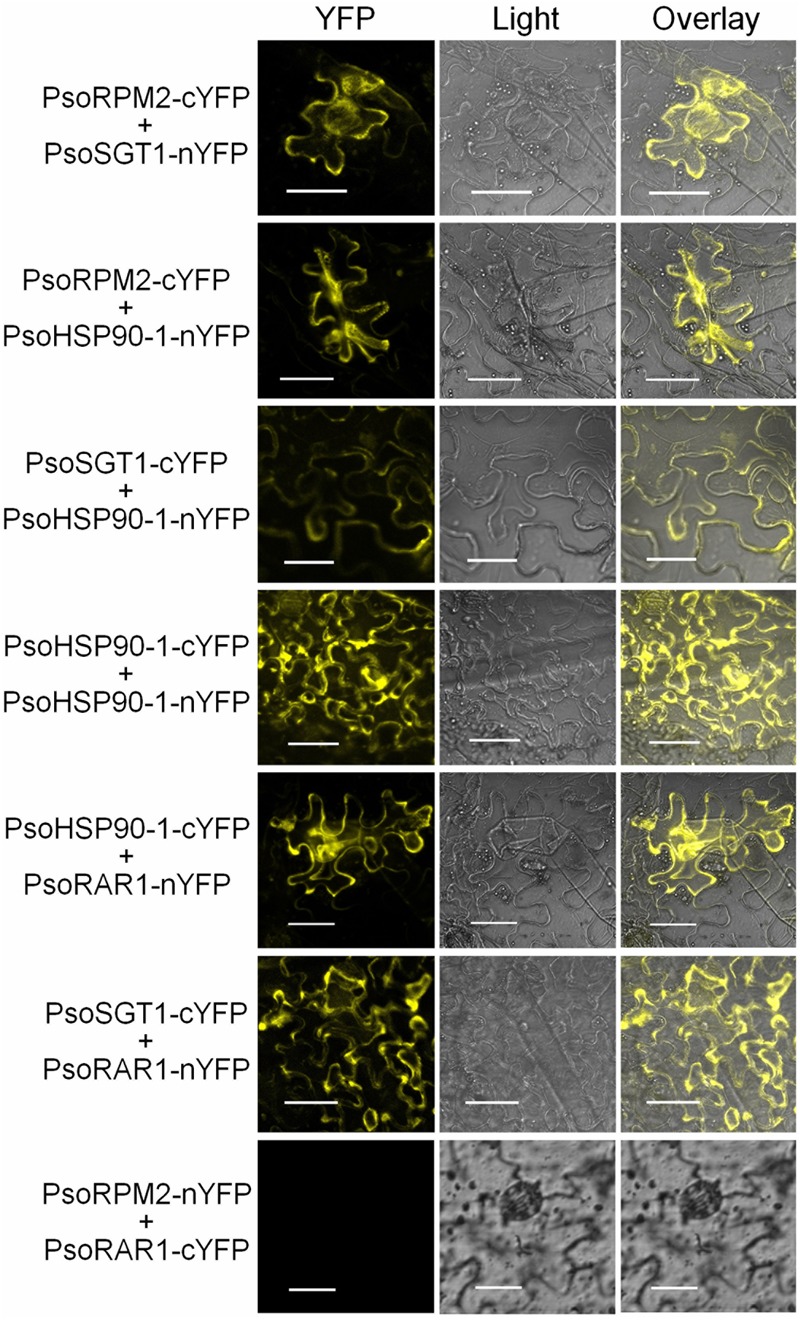
Interactions between PsoRPM2 and its three chaperonin proteins were determined by BiFC. YFP fluorescence in the upper epidermis cells of *N. benthamiana* leaves was detected by laser scanning confocal microscopy. Bar = 40 μm.

To further verify the interactions between PsoRPM2 and its three chaperones *in vivo*, we constructed the bimolecular fluorescent vectors of *PsoRPM2, PsoSGT1, PsoHSP90-1*, and *PsoRAR1* genes. The interaction fluorescence between the PsoRPM2 protein and the three co-chaperone proteins was observed in tobacco leaf epidermal cells via confocal microscopy, using a PsoRPM2-PsoRAR1 combination as negative control. The results showed that the interaction between the PsoRPM2 protein and different chaperone proteins was consistent with the results of the yeast two-hybrid assay. A combination of PsoRPM2-PsoSGT1, PsoRPM2-PsoHSP90-1, PsoSGT1-PsoHSP90-1, the PsoHSP90-1 homodimer, PsoRAR1-PsoHSP90-1, and PsoRAR1-PsoSGT1 was observed, whereas the combination PsoRPM2-PsoRAR1 still did not interact (**Figure 7**).

## Discussion

Several methods for the prevention and control of RKN disease have been developed due to the severity of resulting damage to the agricultural industry; however, it remains a mainstream choice to seek naturally RKN-resistant *R* genes in plants. Previous studies have shown that the *R* gene initiates the identification of pathogen invasion and maintains a resting state if the pathogen is invaded (Jacob et al., 2013). This could reduce the cost of labor and pesticides compared to human intervention. However, the detection of novel *R* genes in plants often requires prior genomic data or prolonged hybridization to obtain resistance-related molecular markers. All this is more difficult to achieve in woody plants, especially in wild fruit tree germplasm resources. Fortunately, previous research has found that most of the *R* genes are highly conserved in their NB-ARC domains (Jacob et al., 2013). This conserved domain can be utilized as a molecular marker (Gupta and Rustgi, 2004), which makes it possible to clone novel *R* genes from plants without prior genomic data such as in the case of the Xinjiang wild myrobalan *P. sogdiana*. A number of resistant genes have been obtained via this RGAs homologous cloning method, such as *MbR4* (Lee and Lee, 2005), *Lr1* (Cloutier et al., 2007), and *GHNTR1* (Zhang et al., 2015). This method is fast and causes low cost; however, it still has several shortcomings. For example, some *R* genes with considerable variability in the NB-ARC conserved region cannot be cloned via homologous (Liu et al., 2014). The *PsoRPM2* gene can rapidly and significantly increase its expression in response to *M. incognita* invasion and thus enhance the RKN resistance of transgenic tobacco lines, suggesting that it is likely involved in disease resistance, which is similar to previous studies on the *Mla* gene (Halterman et al., 2003) and the *GHNTR1* gene (Zhang et al., 2015) against powdery mildew and RKN, respectively.

The PsoRPM2 protein belongs to the TIR-NB-ARC-LRR subfamily, which is similar to *Ma* (Claverie et al., 2011), and the Mi-1 and the GHNTR1 proteins belong to the CC-NB-ARC-LRR subfamily (Milligan et al., 1998; Zhang et al., 2015). The difference between CC and TIR domains may affect the *R* gene function during plant immunity (Shirasu, 2009). The NB-ARC domain in the middle of R proteins has been suggested to act as an elicitor, which may maintain an intramolecular interaction with the LRR domain during the inactive protein state (Tameling et al., 2002; Steinbrenner et al., 2015). The NB-ARC domain structure of the PsoRPM2 protein was most similar to the ced-4 protein (Yan et al., 2004), which was obtained via three-dimensional structure analysis (confidence 100%, coverage 96%). The ced-4 protein mediated apoptosis (Yan et al., 2004), indicating that the NB-ARC domain may achieve the nematode resistance signal of PsoRPM2. This indicated that the NB-ARC domain of PsoRPM2 may induce RKN resistant signaling. The number of repeats of the LRR domain differs between different nematode proteins and its 3D structure was predicted to have a horseshoe structure (Takken and Goverse, 2012). This domain of PsoRPM2 may be associated with pathogen recognition and promotion of disease resistance as previously reported (Krasileva et al., 2010).

The gene function of wild-planted resources is relatively difficult to determine because the genetic transformation systems of these wild germplasms are largely immature or require several years of hybridization. Therefore, it forms a viable alternative approach to use a model plant material without nematode resistance to analyze candidate RKN-resistant genes. In our *PsoRPM2* transgenic tobacco lines, L1–4 were associated with a remarkable reduction of *M. incognita* susceptibility (**Figure 5**). However, the level of RKN resistance varied between the four *PsoRPM2*-expressing transgenic lines. L1 displayed almost no nematode infection symptoms and has the highest expression of the four transgenic lines (**Figure 3B**), while the remaining three transgenic lines exhibited slight signs of nematode infection (**Figures 5D,E**). The expression changes of *PsoRPM2* during nematode infection (0–5 dpi) in transgenic tobacco lines were not obvious (**Figure 3B**), suggest the 35S promoter may not infected by RKN infection. However, even the most RKN resistance L1 did not reach the resistance level of resistant *P. sogdiana* (Li et al., 2011). This demonstrated that xenogeneic transgene method has several shortcomings: several of the extreme examples of heterologous genes may be affected by the host plant immune system during gene expression or protein translation, or the foreign gene cannot properly match the local immunity signaling pathway. An extreme example is that the tomato *Mi-1* gene is neither resistant to RKN nor to the potato aphid when it is expressed in the eggplant (Goggin et al., 2006). A further example is that host genetic background and dose-dependency may influence the *Mi-1* RKN resistance in tomato varieties (Jacquet et al., 2005). In combination with the expression of the *PsoRPM2* gene in resistance *P. sogdiana* and the resistance to RKN of transgenic tobaccos (i.e., L1, **Figures 3B, 5**), we found that the higher expression of the *PsoRPM2* gene usually induced stronger RKN resistance, which is consistent with a previously reported observation for *Mi-1.2* transgenic tomatoes (Milligan et al., 1998). In addition, we found that the *PsoRPM2*-expressing transgenic tobacco lines all exhibited early flowering, which implies that *PsoRPM2* is also involved in plant reproductive development.

Previous studies have shown that the R protein is not only a single promote element in the immune systems of herbaceous model plants, but also requires assistance of the chaperone complex to promote disease resistance (Shirasu, 2009; Tran et al., 2017). With the help of genomic data of *P. mume*, we found that PsoRPM2 and its co-chaperones HSP90, SGT1, and RAR1 could also have interacted (**Figures 6, 7**), suggesting that the cooperative relationship of this R protein and its co-chaperone complex is also present in woody fruit tree. The sequence conservation of these chaperone proteins of different plants suggests that their function may be important for the immunity and physiological processes of plants (Kadota et al., 2010). A lack of HSP90, SGT1, or RAR1 proteins affects the resistance of many *R* genes including the RKN-resistant gene (Hubert et al., 2003; Bieri et al., 2004; Holt et al., 2005; Botër et al., 2007; Van Ooijen et al., 2010), while the SGT1 protein assists the HSP90 protein to maintain a safe R protein level, thus preventing an autoimmune response (Hahn, 2005; da Silva et al., 2007). Similar to *Arabidopsis*, multiple HSP90 homologues exist in *P. mume* and *P. sogdiana*; however, only the PsoHSP90-1 protein interacts with the PsoSGT1 protein (**Figure 6**). This unique manifestation is consistent with an effect observed in *Arabidopsis* (Takahashi et al., 2003). However, it is worth noting that HSP90.2 and HSP90.3 proteins are still involved in pathogen resistance in *Arabidopsis* (Krishna and Gloor, 2001), even though they do not interact with the SGT1-RAR1 protein, indicating redundancy in the function of the HSP90 protein. This suggests that HSP90-2 and HSP83 may also be involved in the disease resistance process of *P. sogdiana*. The PsoHSP90-1 and SGT1 genes are able to form dimers, which is consistent with the findings of previous reports (Nyarko et al., 2007; Röhl et al., 2013). Several R proteins can form homodimeric (Mestre and Baulcombe, 2006; Bai et al., 2012) or heterodimeric interactions (Williams et al., 2014) thus triggering immunity. However, in this study, no interaction was observed in the PsoRPM2 protein (**Figure 6B**), and this complex variation in the plant immune system may be associated with a different disease resistance pattern of the *R* gene. In the limited study of chaperone protein complexes in RKN-resistance publications, the normal function of Mi-1 requires the interaction of Hsp90-1 and Sgt1-1 proteins, whereas RAR1 had no effect on RKN resistance (Bhattarai et al., 2007). PsoRPM2-HSP90-1-SGT1-RAR1 may also possibly have formed a complex in *P. sogdiana*, and the resistant mechanism of the *PsoRPM2* gene may be similar to other *R* genes that require assistance from the co-chaperone protein complex (Kadota et al., 2010).

In summary, our study showed that the *PsoRPM2* gene enhanced the plant RKN-resistance and interacted with its chaperone protein complex. Future work should focus on the resistance mechanism of the PsoRPM2-HSP90-1-SGT1-RAR1 complex during RKN infection and should develop stable genetic transformation systems in *P. sogdiana*.

## Author Contributions

Conceived and designed the experiments: XZ and JH. Performed the experiments: XZ, KX, and HC. Analyzed the data: XZ and KX. Wrote the manuscript: XZ, KX, and JH.

## Conflict of Interest Statement

The authors declare that the research was conducted in the absence of any commercial or financial relationships that could be construed as a potential conflict of interest.

## Footnotes

1 http://blast.ncbi.nlm.nih.gov/Blast.cgi?CMD=Web&PAGE_TYPE=BlastHome

2 http://www.sbg.bio.ic.ac.uk/phyre2/html/page.cgi?id=index

3 http://www.ncbi.nlm.nih.gov/genome/?term=Prunus+mume
