# Supplementary material for: Overexpression of the Prunus sogdiana NBS-LRR Subgroup Gene PsoRPM2 Promotes Resistance to the Root-Knot Nematode Meloidogyne incognita in Tobacco
Source: Front Microbiol. 2017 Oct 31;8:2113. doi: 10.3389/fmicb.2017.02113 (PMC5671597; doi:10.3389/fmicb.2017.02113)
Supplement: Supplementary file 1 [file Table_1.DOCX]

Table S1. Primers used for cloning, expression analyses and vector constructions.

| Name | Sequence 5’-3’ | Application |
| --- | --- | --- |
| MP-F | GGNATGGGNGGNDTNGGNAARACNAC | Includes P-loop motif for RGAs |
| MP-R | NACYTTNARNGCNARNGGNARNCC | Includes hydrophobic motif for RGAs |
| PsoRPM2-GSP1 | GCTGAAATCAACAAGAAACCGACCAAAT | Special primers for RACE |
| PsoRPM2-GSP2 | CGGTTCCAGAAATGAATCCAGATGAAGG |  |
| PsoRPM2-GSP3 | ATTCTACTTCCAGAGCCAAACCAATCAC |  |
| PsoRPM2-GSP4 | TTATCATACCCATCCCTCCATCAACAC |  |
| UPM-long | CTAATACGACTCACTATAGGGCAAGCAGTG GTATCAACGCAGAGT | Universal primers for RACE |
| UPM-short | CTAATACGACTCACTATAGGGC |  |
| PsoRPM2-qPCR-F  PsoRPM2-qPCR-R | TGCACCAATCTCACCACT | Expression analysis by RT-qPCR |
|  | CCGAAGAGTAAACGCAAC |  |
| RPII-F  RPII-R  NtActin-F  NtActin-R | TGAAGCATACACCTATGATGATGAAG |  |
|  | CTTTGACAGCACCAGTAGATTCC |  |
|  | TTGCCTGATGGACAAGTTATTACC |  |
|  | TAGGAGCCAAAGCCGTGATT |  |
| PsoRPM2-F | CGAAACAAGTCGTTCAGTTC | Clone full-length of corresponding gene ORFs |
| PsoRPM2-R | CCTAATTGAATACTACCATA |  |
| PsoHSP90-1-F | CATCTGTTAGGTTTAGGGTTC |  |
| PsoHSP90-1-R | CCAAGTTAGCGACCGTATT |  |
| PsoHSP90-2-F | CACTACCTCCTCTGTCTTCA |  |
| PsoHSP90-2-R | AACAGCCGTAGGAGACAAT |  |
| PsoHSP83-F | TTCTTCTGCTTCGCTTTCT |  |
| PsoHSP83-R | CAAATCCAATTTACACCTCAA |  |
| PsoSGT1-F | AGAGTCAGTCACCGTGCTTTGC |  |
| PsoSGT1-R | GAGTGCCAGACGACTTGGAAAA |  |
| PsoRAR1-F | GCTTATTGTTTTGCATGT |  |
| PsoRAR1-R | TGTAGGACCAAAAGGAGA |  |

| PsoRPM2-ORF-F | CATGCCATGGATGGGCTCCGGGGC | Construct and test victors for *PsoRPM2* transgenic analyses |
| --- | --- | --- |
| PsoRPM2-ORF-R | GACACAGGCTAGCAAACAATCCATTAA |  |
| GFP-F | GACACAATGGTGAGCAAGG |  |
| GFP-R | GGACTAGTTTACTTGTACAGCTCGTCCA |  |
| HPT-F | CCTGAACTCACCGCGACG |  |
| HPT-R | AAGACCAATGCGGAGCATATAC |  |
| PsoSGT1-AD-F | GGAATTCCATATGATGGCTTCCGATCTC | Construct victors for yeast two-hybrid analyses |
| PsoSGT1-AD-R | CCGCTCGAGTTAGTACTCCCATTT |  |
| PsoSGT1-BK-F | CATGCCATGGCTTCCGATCTCGAAA |  |
| PsoSGT1-BK-R | ACGCGTCGACTTAGTACTCCCATTT |  |
| PsoRPM2-ABD-F | CGGAATTCATGGGCTCCGGGGCA |  |
| PsoRPM2-AD-R | CGAGCTCAAACAATCCATTAAA |  |
| PsoRPM2-BK-R | ACGCGTCGACAAACAATCCATTAAA |  |
| PsoHSP90-1-ABD-F | CGGAATTCATGGCTCCAGTTCTA |  |
| PsoHSP90-1-AD-R | CGAGCTCTCAGTCGCTCCATGGATCG |  |
| PsoHSP90-1-BK-R | ACGCGTCGACTCAGTCGCTCCATGG |  |
| PsoHSP90-2-AD-F | CGGAATTCATGGCGGAGACTGAG |  |
| PsoHSP90-2-AD-R | CCGCTCGAGTTAATCAACTTCCTC |  |
| PsoHSP90-2-BK-F | CATGCCATGGCGGAGACTGAG |  |
| PsoHSP90-2-BK-R | CGGAATTC TTAATCAACTTCCTC |  |
| PsoHSP83-ABD-F | GGAATTCCATATGGCGGACGTTCA |  |
| PsoHSP83-ABD-R | TCCCCCGGGTTAGGCGTCCTCCTC |  |
| PsoRAR1-ABD-F | GGAATTCCATATGGAGGGTCAGCAG |  |
| PsoRAR1-ABD-R | TCCCCCGGGTTAAGATACTGGAT |  |
| PsoHSP90-1-Inj-F | GCGTCGACATGGCTCCAGTTCTA | Construct victors for BiFC analyses |
| PsoHSP90-1-Inj-R | GGACTAGTGTCGCTCCATGGATC |  |
| PsoSGT1-Inj-F | GCGTCGACATGGCTTCCGATCT |  |
| PsoSGT1-Inj-R | GGACTAGTGCACTCCCATTTCTTCA |  |
| PsoRAR1-Inj-F | CGGGATCCATGGAGGGTCAG |  |
| PsoRAR1-Inj-R | GGACTAGTAGATACTGGATCAG |  |
| PsoRPM2-Inj-F | GCGTCGACATGGGCTCCGGGGCA |  |
| PsoRPM2-Inj-R | GGACTAGTAAACAATCCATTAAA |  |

*Underlined part of the primers indicates restriction enzyme cutting site and protective bases.
